# Acceptance and self-reported use of a dementia care toolbox by general practice personal: results from an intervention study in German practices

**DOI:** 10.1186/s12875-020-01345-0

**Published:** 2020-12-09

**Authors:** Anna-Liesa Filbert, Sabine Christine Jäger, Birgitta Weltermann

**Affiliations:** Institute of General Practice and Family Medicine, University of Bonn, Venusberg-Campus 1, 53127 Bonn, Germany

**Keywords:** Dementia, Dementia care, General practitioner, General practices, Family medicine, Migration background

## Abstract

**Background:**

Dementia is an age-related syndrome that is estimated to affect 46.8 million people worldwide (2015). In ageing populations, the prevalence of dementia is expected to increase. General practitioners (GPs) are often the first to be contacted when signs of dementia appear. This cluster-randomised trial (CRT) investigates the effects of a dementia care toolbox mailed to GP practices to facilitate dementia care. It contained patient brochures and posters for the waiting room in three languages, information cards for professionals and practical tools in three languages. The GPs’ and practice assistants’ (PrAs) use of and opinion about the toolbox is reported here.

**Methods:**

Three months after receiving the toolbox, participating GPs and PrAs were sent a standardised, self-administered questionnaire asking about the use and helpfulness of the various toolbox items by mail.

**Results:**

A total of 50 GPs and PrAs (14 GPs and 36 PrAs) from 15 practices completed the questionnaire. Of the participants, 82.0% reported using at least one of the tools, while 18.0% had used none. In descending order, the patient brochures (70.0%), the information card (58.0%) and the poster (40.0%) were used. In general, the brochures (52.1%), the information card (44.9%) as well as the poster (28.6%) were perceived as helpful.

**Conclusion:**

Overall, the dementia toolbox was widely accepted by both professional groups. Future research should investigate long-term effects of information strategies for GP practice settings.

**Trial registration:**

German Clinical Trials Register, DRKS00014632. Registered 02 August 2018. Clinical register of the study coordination office of the University hospital of Bonn. Registered 05 September 2017*.*

## Background

Dementia has become an increasing challenge worldwide especially among the rapidly growing elderly population. With an estimated prevalence of 46.8 million, it is expected that 131.5 million people will suffer from dementia by 2050 [1]. As no specific treatment exists yet, it is ranked the fifth most common cause of death worldwide [2, 3].

Dementia is age-related and describes a progressive neurodegenerative disorder. It is characterised by a decline in memory and cognitive deficits in learning ability, concentration and orientation persisting for at least 6 months [4]. These cognitive deficits gradually impair the affected persons’ ability to perform activities of daily living. In Germany, 1.2 million people suffer from dementia, Alzheimer’s Disease (AD) being the most common subtype [5]. AD is a neuropathological disorder characterised by an abnormal cerebral accumulation of intra-neutral hyper-phosphorylated tau protein (p-tau) and extra-neural beta-amyloid plaques (Aß) causing cerebral atrophy [3]. Vascular dementia (VD), which accounts for about 10% of dementia cases, results from micro or macro brain strokes due to damages or blockages of cerebral vessels [5, 6]. However, AD and VD often coexist and cannot always be separated [5]. Research has shown that the prevalence of dementia after a first stroke increases to 10% and even exceeds 30% after stroke recurrences [7]. Therefore, in order to lower the overall risk of dementia it is important to prevent recurrent strokes and to manage prominent cerebrovascular risk factors associated with AD and VD such as hypertension, atrial fibrillation, diabetes, hypercholesterolaemia, lack of physical activity and obesity [8].

Because dementia progresses slowly over many years from an asymptomatic stage to a full clinical manifestation, and early symptoms are often misinterpreted and advanced symptoms are recognised only in the late stage, diagnosing the disease is challenging [9]. This is reflected in the fact that mild dementia is often underdiagnosed [10]. However, there is an urgent need for early diagnosis in order to ensure the earliest possible access to treatment options and adequate intervention programs for patients and to support caregivers [10–12]. Moreover, keeping patients in their familiar environment, reducing feelings of anxiety and uncertainty, improving quality of life and independence as well as reducing psychological distress have all been shown to be beneficial for patients, as it gives them more time to cope with the diagnosis [13–15]. In Germany, general practitioners (GPs) are usually the first point of contact when signs of dementia occur. However, overall there has been a decline in the number of general practitioners [16]. The situation is further complicated by the fact that GPs are confronted with challenges due to demographic change and multimorbidity [17].

Several studies among GPs worldwide have shown that the above-mentioned difficulties are due to lack of confidence in the diagnosis, time, case complexity, fear of early labelling and uncertainty about which instruments and tests are useful [18–21]. Training on the use of testing tools was therefore considered useful especially for young professionals [19, 20]. Providing materials in different languages to improve the diagnosis of dementia in migrants is also considered expedient [19, 20].

In order to facilitate dementia care in German general practices for patients with and without a migrant background, the effects of a toolbox consisting of four tools for GPs and practice assistants (PrAs) were investigated. The intervention group was asked about their use and perceived helpfulness of the toolbox.

## Methods

### Study design

**Fig. 1:**
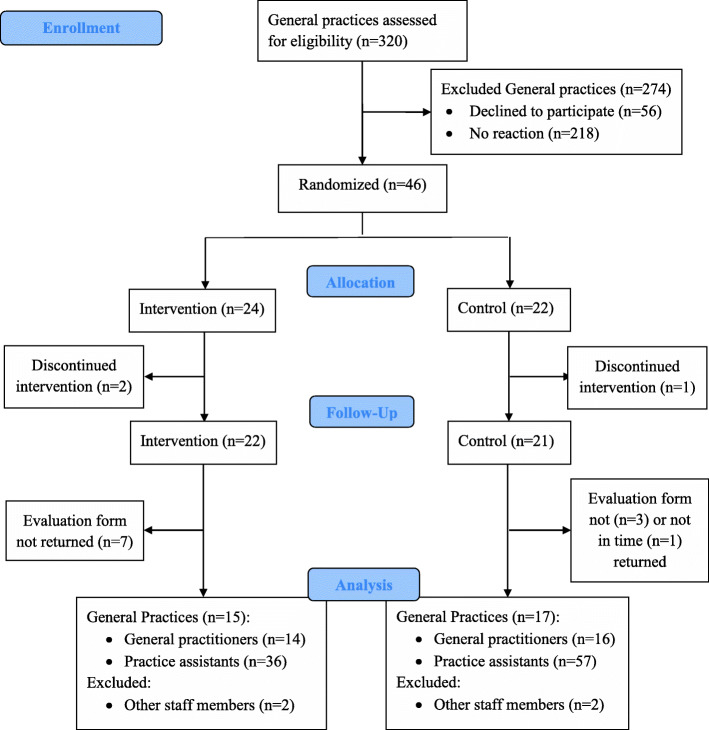
Flow diagram showing the selection process of the study participants

A cluster-randomised, wait list-controlled intervention study was conducted on the basis of the previously published exploratory baseline survey, which showed that GPs were interested in receiving further information on how to deal with dementia patients [22]. Thirty-two out of 320 general practices across North Rhine-Westphalia in Germany were recruited as shown in Fig. 1. The study was conducted between September 2018 and April 2019. Practices were randomly allocated to receive the dementia care toolbox at baseline or after 3 months.

A standardised, self-administered, written evaluation survey was used to ask for details relating to confidence and/or professional dementia care, dementia care in the last 3 months and opinions on education material with its perceived effect. In addition, GPs and PrAs were asked to provide their sociodemographic data, including age, sex, language skills, duration of employment and migrant background. The study was registered with the German Clinical Trials Register (DRKS) (DRKS-ID: DRKS00014632) and the clinical register of the study coordination office of the University Hospital Bonn [23].

### Participants

The target group of this study were GPs and their PrAs in the North Rhine region. Two hundred thirty practices from the previous random sample in the cross-sectional study as well as 90 practices from the Institute’s network of teaching physicians or practices from which a research interest was known, were contacted [22]. Actively practicing practices whose physician was registered as a contract physician in the database of the Association of Statutory Health Insurance Physicians of North Rhine-Westphalia (KVNO) on 28 July 2017 and was listed in the KVNO database as a family physician were included.

Physicians with the designation paediatrician in the KVNO database were excluded.

### Intervention and control conditions

After obtaining written informed consent for study participation by fax or mail, the information material was sent by mail to the GP practices randomised to the intervention group. At the same time, the practices of the control group received a short letter on the further procedure. The intervention material was then used for 3 months, following which the physicians and PrAs of the intervention and control groups were interviewed by means of a standardised, self-administered evaluation form.

### Intervention condition: toolbox

The toolbox materials were developed according to the needs identified by the GPs in a prior baseline survey and the experience of regional contact points for dementia patients and their next of kin obtained via telephone contact. Furthermore, the practical tools of the toolbox contain already existing materials so that the composition of the individual components is newly developed. The baseline survey documented that GPs experience language barriers in the diagnosis of dementia, especially among patients with a Turkish or Russian background [22]. This result was plausible, as migrants from European countries account for 30.4% of the population in the North Rhine region, with most of them coming from Turkey (5.34%) and Russia (2.18%) [24].

The toolbox consisted of four different materials (Table 1):

1. Brochures: The eight-page brochure was written for patients and their next of kin in three languages (German, Turkish, Russian) to sensitise them for dementia. In addition to a definition of dementia, it included typical symptoms and a description of the general procedure after first symptoms appear. Moreover, contact addresses (website and telephone numbers) of support services for patients and their next of kin as well as contact points specifically for people with a migrant background were provided. The brochure was written in a common, understandable language, and symptoms as well as the further steps taken by the physicians were graphically illustrated in the form of symbols and diagrams.
2. Poster: The poster (30 cm × 42 cm) with the headline “Are you or your next of kin familiar with these situations?” was provided for the practices’ waiting rooms. The aim was to make patients and their next of kin aware of typical symptoms of dementia and to encourage them to consult their GP if any such symptoms occurred. The symptoms were presented in the form of short questions and statements, such as “Where am I?” and “I often misplace objects”. Additionally, these sentences were supported by the same symbols as in the brochure. The original German poster was translated into Turkish and Russian.
3. Information card: The target group of the information card were the GPs and their PrAs. The double-sided printed card was written in German. In addition to a definition of migrant background and epidemiological facts on dementia diagnostics, it contained assistance on the following aspects: challenges, communication advice, support services in case of language problems, cultural sensitivity and contact addresses for dementia patients with and without a migrant background. For example the paragraph on support services in case of language problems includes tips such as online translators or a homepage on which it is possible to search for GPs according to language skills.
4. Practical tool: The practical tool consisted of three parts: a double-sided printed medical history sheet in Turkish/German, English/German and Russian/German (supplied by Setzer Verlag), the standardised EASY test, a non-verbal, culture-fair screening procedure for the detection of cognitive impairment, and a booklet. The booklet “Diversity in the practice” published by the National Association of Statutory Health Insurance Physicians was given to GPs in German and included information on health competence and tips for communication, especially for patients with a migrant background. In addition to examples and tips for cultural sensitisation, the booklet contained various interviews with specialists, legislation for treatment and further service addresses. The booklet consisted of 20 pages and was designed with pictures, diagrams, symbols, highlighted headlines as well as subheadings.

**Table 1:** Description of the intervention toolbox

Item	Target group	Aim of material	Topic/Content	Layout	Language
8-page brochure	Pat, next of kin	Provide overview and support	• Definition of dementia and symptoms • Contact addresses • Procedural steps (GPs)	• Symbols • Diagram • Highlighted keywords • Websites	Common language: Ger, Rus, Tur
Poster (30 cm × 42 cm)	Pat, next of kin	Creating awareness	• Questions about key symptoms of dementia	• Symbols	Common language: Ger, Rus, Tur
2-page information card	GP, PrA	Information on how to deal with patients with and without a migrant background	• Support services in case of language problems • Cultural sensitivity • Contact addresses • Communication advice	• Symbols • Highlighted keywords • Websites	Ger
Practical tool	GP	Facilitation of diagnostics for people with and without a migrant background	• Medical history sheet • EASY test • 20-page booklet	• Highlighted keywords • Symbols • Interviews • Diagrams • Websites	Ger-Eng Ger-Tur Ger-Rus

Target group: *GP* General Practitioner, *Pat* Patients, *PrAs* Practice Assistants, Language: *Eng* English, *Ger* German, *Tur* Turkish, *Rus* Russian

### Control condition

Practices randomised to the control group did not receive any intervention, i.e. treatment as usual. After the follow-up data were collected in the intervention and control groups, the practices in the waiting-list control group received the toolbox.

### Data collection

After the toolbox was used in the intervention group for 3 months, all practices (intervention and control group) received the evaluation survey. GPs and PrAs completed the form to assess the acceptance and use of the toolbox materials and returned it to the study coordinator. Reminders to complete and return the questionnaire were given once in writing and twice by telephone.

The questionnaire used at follow-up comprised a total of 13 questions supplemented by eight questions for the intervention group only. For comparison, about a third of the questions were identical to the questionnaire, which was used for our prior cross-sectional survey [22, 25]. The additional questions addressed the acceptance and use of the toolbox, e.g. role/duties in the practice, how they used/liked the toolbox materials, and estimated frequency of contacts to dementia care patients/migrants in last 3 months.

The physicians’ data were pseudonymized; those of the PrAs were anonymous.

For the purposes of this paper, the answers to the following questions translated in German were included in the descriptive analyses of the intervention group. The answer categories were 5-point Likert scales ranging from “not helpful” to “very helpful” (a), multiple answers with or without additional free text fields (b) as well as closed questions (yes/no) (c):

To what extent did you use the information material provided?^(b)^

How helpful do you find the information card that was developed for you?^(a)^

Which sections of the information card do you find particularly helpful?^(b)^

How helpful do you find the poster for your patients/family?^(a)^

How helpful do you find the information brochure for your patients/family members?^(a)^

Do you find the practical material (multilingual medical history sheets, EASY short test, booklet) helpful?^(c)^

### Data management and statistical methods

The questionnaires were scanned using the TeleForm data capture system. Extracted data were checked visually through a comparison with the original questionnaires. All data were analysed using descriptive statistics in IBM SPSS-Statistics for Windows, version 25. All above mentioned questions answered by GPs and PrAs were included in the analysis whereas missing data were not considered. Absolute and relative frequencies, means and standard deviations are reported for valid cases. To control for confounding, descriptive statistics adjusted for age, gender, duration of employment in this family practice and migration background were computed for all participants.

## Results

The distribution of participants varied between one and six in the 15 practices. For a detailed distribution, see Table 2.

**Table 2:** Distribution of participating GPs and PrAs among the individual practices

Practices	Intervention group	GP	Medical assistant
1	2	1	1
2	2	1	1
3	6	1	5
4	3	1	2
5	2	1	1
6	2	1	1
7	4	1	3
8	6	1	5
9	3	1	2
10	1	1	0
11	1	1	0
12	3	0	3
13	6	1	5
14	5	1	4
15	4	1	3

### Characteristics of the study population

A 10.0% response rate was achieved. Thirty-two practices, 15 of which were randomised to the intervention group and 17 to the control group, returned the questionnaire. The following analyses considered only the data of the intervention group, which consisted of 14 GPs (28.0%) and 36 PrAs (72.0%). Of the 14 family doctors, 71.4% were male and 28.6% were female. Among the PrAs the distribution was 97.2% female and 2.8% male. The participants’ mean age was 45.6 years (SD ± 13.7), 55.6 years (SD ± 6.5) in GPs and 41.5 years (SD ± 13.8) in PrAs. If the age is dichotomized into < 50 years (50.0%) and > =50 years (50.0%), the age distribution was balanced. The average duration of employment in this family practice was 16.7 (SD ± 11.4) years. Among the participating GPs, two (14.2%) indicated having a migration background, and five (14.7%) among the PrAs. Considering the number of languages apart from German that can be spoken fluently, 13 (40.6%) of the PrAs did not speak any, which is not the case for any of the GPs. 17 (53.1%) PrAs spoke one foreign language fluently, two (6.3%) PrAs spoke two foreign languages and none of the PrAs spoke three foreign languages. Among the GPs, six (42.9%) spoke one foreign language fluently, four (28.6%) spoke two and another four (28.6%) spoke three foreign languages fluently. Characteristics of the study population are summarised in Table 3.

**Table 3:** Characteristics of the study population

	Total study population		Intervention group	
	n	(%)^**a**^	n	(%)^**a**^
**Total participants**	123	(100)	50	(40.6)
**Total practices**	32	(100)	15	(46.9)
	**n**	**(%)**^**a,b**^	**n**	**(%)**^**a,b**^
**Profession**				
GPs	30	(24.4)	14	(28.0)
PrAs	93	(75.6)	36	(72.0)
**Gender**				
Female	103	(83.7)	39	(78.0)
Male	20	(16.3)	11	(22.0)
**Age**				
< 50	67	(54.5)	25	(50.0)
> =50	56	(45.5)	25	(50.0)
**Duration of employment in this family practice**				
< =5 years	29	(24.0)	12	(24.5)
< =15 years	40	(33.1)	14	(28.6)
> 15 years	52	(43.0)	23	(46.9)
**Was your mother or father or were you yourself born abroad?**				
Yes	19	(15.7)	7	(14.6)
No	102	(84.3)	41	(85.4)
**Which languages apart from German do you speak fluently that you are able to treat a foreign-language patient in your family doctor’s practice?**				
0	30	(26.3)	13	(28.3)
1	59	(51.8)	23	(50.0)
2	19	(16.7)	6	(13.0)
3	6	(5.3)	4	(8.7)

^a^ Column percentages

^b^ Percentages are reported for valid cases

### Use of intervention material

**Fig. 2:**
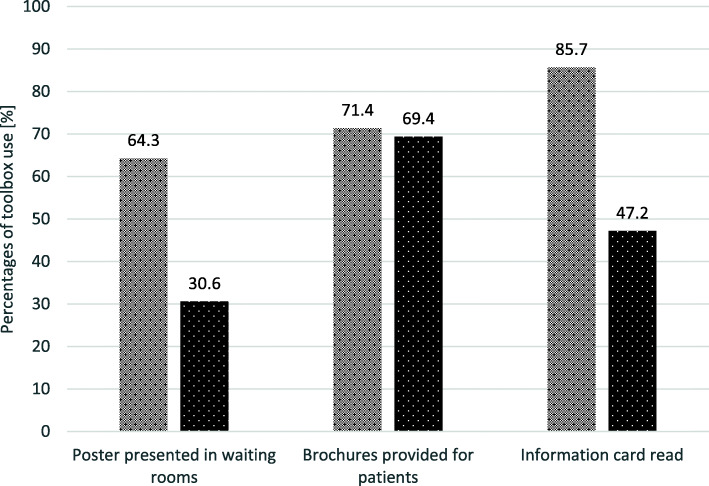
GPs’ and PrAs’ use of the toolbox materials

Overall, GPs used the toolbox materials more often than PrAs. 82.0% of the participants used at least one of the four intervention tools, whereas 18.0% did not use any. 30.0% of the participants stated that they had used three out of four information materials. No participant applied all four information tools (Table 4). In descending order, the brochures (70.0%), the information card (58.0%) and the poster (40.0%) were used. 70% of both professional groups used the brochures. More precisely, GPs used the information card (85.7%) most often and the poster (64.3%) the least. In comparison, PrAs used the brochures most often (69.4%) and the posters (30.6%) the least. 85.0% of GPs, but only about half of PrAs, used the information card (Fig. 2).

**Table 4:** Information materials used by the study participants with different functions

	Intervention group		GPs		PrAs	
	n	(%)^**a**^	n	(%)^**a**^	n	(%)^**a**^
**Quantity of intervention material used**						
0	9	(18.0)	2	(14.3)	7	(19.4)
1	13	(26.0)	1	(7.1)	12	(33.3)
2	13	(26.0)	3	(21.4)	10	(27.8)
3	15	(30.0)	8	(57.1)	7	(19.4)
4	0	(0)	0	(0)	0	(0)

^a^ Column percentages

### Opinion about the intervention material

In descending order, the brochures (52.1%), the information card (44.9%) and the posters (28.6%) were considered helpful. More precisely, the following aspects, listed in descending order, were considered helpful: support for patients with and without a migrant background (41.7%), support services in case of language problems (27.1%), communication advice (22.9%), cultural sensitivity (6.3%) and challenges (4.2%). GPs as well as PrAs rated the support for patients with and without a migrant background as most helpful (GPs: 38.5%, PrAs: 42.9%). The least helpful aspect among GPs was support services in case of language problems (7.7%), whereas among PrAs it was support for cultural sensitivity (2.9%) (Table 5).

**Table 5:** Opinions of GPs and PrAs about the dementia care toolbox

	Intervention group		GPs		PrAs	
	n	(%)^**a,b**^	n	(%)^**a,b**^	n	(%)^**a,b**^
**How helpful was the poster?**						
Unhelpful	0	(0)	0	(0)	0	(0)
Slightly helpful	18	(36.7)	5	(38.5)	13	(36.1)
Helpful	14	(28.6)	3	(23.1)	11	(30.6)
Very helpful	3	(6.1)	2	(15.4)	1	(2.8)
*Not applicable*	*14*	*(28.6)*	*3*	*(23.1)*	*11*	*(30.6)*
**How helpful was the brochure?**						
Unhelpful	1	(2.1)	0	(0)	1	(2.9)
Slightly helpful	8	(16.7)	1	(7.7)	7	(20.0)
Helpful	25	(52.1)	6	(46.2)	19	(54.3)
Very helpful	7	(14.6)	3	(23.1)	4	(11.4)
*Not applicable*	*7*	*(14.6)*	*3*	*(23.1)*	*4*	*(11.4)*
**How helpful was the information card?**						
Unhelpful	4	(8.2)	2	(15.4)	2	(5.6)
Slightly helpful	12	(24.5)	3	(23.1)	9	(25.0)
Helpful	22	(44.9)	6	(46.2)	16	(44.4)
Very helpful	3	(6.1)	1	(7.7)	2	(5.6)
*Not applicable*	*8*	*(16.3)*	*1*	*(7.7)*	*7*	*(19.4)*
Which aspect in particular did you find helpful?						
Challenges	2	(4.2)	2	(15.4)	0	(0)
Communication advice	11	(22.9)	4	(30.8)	7	(20.0)
Support services in case of language problems	13	(27.1)	1	(7.7)	12	(34.3)
Support for cultural sensitivity	3	(6.3)	2	(15.4)	1	(2.9)
Support for patients with and without a migrant background	20	(41.7)	5	(38.5)	15	(42.9)

^a^ Column percentages

^b^ Percentages are reported for valid cases

## Discussion

### Key findings and interpretation

The purpose of this study was to facilitate dementia care in German general practices by using a newly developed toolbox. With a setting-related approach, the toolbox addressed different target groups in the GP practice setting (GPs, PrAs, patients and next of kin) and included a wide variety of intervention materials. Overall, the toolbox was well accepted (82.0%) and considered helpful; in particular the brochure was rated as helpful by both professions. Our result was to be expected, as 50–80% of physicians use printed materials to obtain information [26]. This passive use of printed educational materials may be used to improve knowledge, awareness, attitudes and patient outcomes in different settings [26–29]. Although various studies have shown a positive effect of information material in practices on the health of patients, the design, content and writing style are criticised [30, 31]. This shows that the characteristics of the intervention material (source of the information, content and channel through which it is delivered) are of importance with regard to its effectiveness [32]. In this study, brochures for patients and next of kin were found to be helpful by GPs and PrAs alike. In fact, brochures are widely used in patient education and are greatly appreciated by patients as a means of obtaining information on health issues [33]. The same applies to the poster for the waiting rooms and the brochures, which PrAs considered helpful. Both are suitable information strategies for GPs practice, especially PrAs, as they require little effort in a work scenario which tends to be challenging, multitasking and – at least sometimes – stressful for personal [34]. Providing such brochures and posters to patients gives them the opportunity to strengthen their right of self-determination and self-control as well as to make independent decisions on relevant health issues [35]. At the same time, it addresses the desire of many next of kin for more information material [36]. This is of major relevance, as the diagnosis dementia also affects the family and has a significant impact on the future family life. Also, it follows the recommendations of previous studies to involve and support the next of kin in dementia care [32]. As described above, dementia patients also reported that they themselves felt insufficiently informed about the disease [36]. Our intervention materials address this imbalance. Furthermore, as Protheroe et al. (2015) showed in their latest study that three out of four patient information leaflets in general practices were judged too complex to read by 15% of the English population [30], our study paid particular attention to a patient-friendly design, specific content and readability of the material. In our study, this was generally achieved through the use of headlines, bullet points, highlighted key messages, the division of topics into sub-topics, a simple design, the use of common language, specific contact addresses and sources of detailed information.

Regarding the information card for GPs and PrAs, the effectiveness of these design and content elements of the printed educational material are in line with the results of an earlier qualitative study by Grundniewciz et al. (2016) among Canadian GPs [31]. In their study, they investigated physicians’ preferences regarding the design and content of printed educational materials. Physicians preferred short, simple and concise materials with references for more detailed information not least due to lack of time. They also emphasised the significant impact of design and content selection on the perceived usability and actual usability of the materials. Furthermore, physicians use training materials to manage and reduce the diversity of new guidelines and evidence [31]. The fact that PrAs rated the aspect “support services in case of language problems” higher may indicate that they are more often in the situation of not being able to communicate with a patient as desired or required due to language barriers. This might be due to the fact that PrAs speak fewer languages than GPs or that PrAs are often the first contact person for the patient.

As mentioned in the introduction section, this topic is becomingly increasing important due to the ageing population. In general, the diagnosis of dementia should be critically reviewed and after information has been provided to those affected [5, 32]. The principle that there is a right not to know must always be considered [5]. Our materials are intended to provide psychosocial support for patients and families; this is particularly important in the case of dementia, as there is evidence that the integration of psychosocial elements is of similar importance to medication [5, 37, 38].

In general, there is a gap between previous studies on dementia care and the populations studied. Migrants have thus far received little attention in this context, therefore it can be assumed that dementia is underdiagnosed in migrants [39]. Our study has considered this problem by compiling the toolbox material in different languages according to the distribution of migrants in North Rhine-Westphalia.

GPs play a key role in optimising outpatient care, which is of major importance as most dementia patients hope to be able to stay in their familiar environment for as long as possible [40] However, it has been shown that the provision of advice and information via the family doctor is problematic [41].

### Limitations

The key strength of the current study is its physician and practice assistant approach which indirectly addressed patients and families. As our intention was to provide an initial overview of the usefulness and opinion about the newly developed toolbox, the validity and reliability of the self-administered questionnaires were not further investigated. In terms of validity, it should be noted that the answer options for the practical tools differ from the other items in the toolbox and that not all parts of the practical tool were tested individually for usefulness. A response bias cannot be excluded for two reasons: First, it can be assumed that mainly GP practices participated with a general interest in research and/or the topic of dementia. Second, despite our efforts (e.g. reminders), the proportion of non-respondents was high. To counteract the problem of validity, we used an anonymised/pseudonymised self-administered paper questionnaire. We did not obtain any information on the acceptance of the toolbox items on behalf of the end users. Self-reported outcomes and the associated socially desirable responses represent a potential bias. The study addressed acceptance of toolbox materials, the effectiveness of patient-related outcomes needs to be evaluated in future. Due to the small sample size the results need to be interpreted with caution.

## Conclusion

The mere diagnosis of dementia is a threat to the livelihood of the patient and his family. The toolbox helps to avoid feeling alone and closes the gap between the diagnosis and further support.

Further research is needed to identify the long-term effects of information strategies for the setting of GP practices. Our study documented the need for and acceptance of the concept of the dementia care toolbox, especially the information brochures, in the setting of general practitioners’ practices.

## Data Availability

The datasets used and/or analyzed for the current study are available from the corresponding author on reasonable request and with permission of the responsible ethics´ committee.

## Abbreviations

AD: Alzheimer’s disease
Aß: Beta-amyloid plaques
GP: General practitioner
KVNO: Association of statutory health insurance physicians of North Rhine-Westphalia
p-tau: Hyperphosphorylated tau protein
PrA: Practice assistant
VD: Vascular dementia
